# “Stroke - 65 Plus. Continued Active Life”: a study protocol for a randomized controlled cross-sectoral trial of the effect of a novel self-management intervention to support elderly people after stroke

**DOI:** 10.1186/s13063-018-2961-4

**Published:** 2018-11-19

**Authors:** Hanne Pallesen, Erhard Trillingsgaard Næss-Schmidt, Simon Svanborg Kjeldsen, Sedsel Kristine Stage Pedersen, Susanne Lillelund Sørensen, Iris Brunner, Jørgen Feldbæk Nielsen

**Affiliations:** Hammel Neurorehabilitation Centre and University Research Clinic, RM, University of Aarhus, Voldbyvej 15, 8450, Hammel, Denmark

**Keywords:** Self-management, Support, Self-efficacy, Quality of life, Stroke, Elderly people

## Abstract

**Background:**

Elderly people represent the majority of stroke cases worldwide. Post-stroke sequelae frequently lead to a more isolated life. Restricted social relations render older individuals with stroke a vulnerable group, especially in terms of social reintegration. Reintegration into the community after a stroke largely depends on support from the family. However, close relatives are at risk of becoming overburdened. The aim of this study is to investigate the effect of a novel self-management intervention to support elderly people after stroke.

**Methods/Design:**

Randomized controlled trial. Two weeks before discharge from a rehabilitation hospital/center, individuals with stroke aged > 65 years will be randomized either to a group receiving conventional neurorehabilitation (control) or to an additional novel self-management intervention. In the intervention group, patients with stroke will be offered eight self-management sessions of 45–60 min duration by a physiotherapist or an occupational therapist during a period of nine months after discharge. Inclusion will continue until at least 35 individuals in each group have been recruited.

Study outcome measurements: Stroke Self-efficacy Questionnaire, a short version of Stroke Specific Quality of Life Scale, Impact on Participation and Autonomy and Caregiver Burden Scale. Furthermore, physical activity will be assessed using accelerometers. All outcomes except “impact on participation” and “autonomy” will be assessed at baseline, three months, and nine months after discharge. Impact on participation and autonomy will be assessed at three and nine months after discharge.

Patient, informal caregiver, and therapist satisfaction will be examined by way of questionnaires and interviews.

**Discussion:**

Self-management interventions are promising strategies for rehabilitation, potentially increasing self-efficacy, quality of life, as well as participation and autonomy. The introduction of a novel self-management intervention in combination with traditional physical and occupational therapy may enhance recovery after stroke and quality of life and lessen the burden on relatives. This trial “Stroke - 65 Plus. Continued Active Life,” will provide further evidence of self-management strategies to clinicians, patients, and health economists.

**Trial registration:**

ClinicalTrials.gov, NCT03183960. Registered on 12 June 2017.

**Electronic supplementary material:**

The online version of this article (10.1186/s13063-018-2961-4) contains supplementary material, which is available to authorized users.

## Background

Stroke often results in physical, psychological, cognitive, and behavioral difficulties, which may cause an unexpected interruption to normal life cycle [1, 2]. Life expectancy is still increasing worldwide. Ageing populations will lead to a higher incidence of strokes, since the risk of suffering a stroke increases with age [3]. Combined with an increased number of stroke survivors [3], many societies are facing major challenges—the workload/financial burden on services—in stroke rehabilitation.

Post-stroke sequelae lead to a more isolated life five years after the stroke [4, 5]. Restricted social relations render elderly people with stroke an especially vulnerable group, in terms of social reintegration [6, 7]. Reintegration into the community after stroke largely depends on support from family members [7, 8]. However, close relatives may be overburdened and at risk of developing anxiety and depression [9].

Evidence from previous studies suggests that self-management programs may be beneficial for people living in the community after a stroke [7, 10, 11]. Broadly, the term self-management focuses on those actions individuals and others take to mitigate the effects of a long-term condition and to maintain the best possible quality of life [12]. Fryer et al. have found that stroke patients might improve self-efficacy and quality of life by taking part in such programs [10]. Furthermore, increased activity after discharge is associated with increased quality of life [2, 13–15]. In two qualitative studies, people with stroke living in the community describe self-management as an important, vital feature of life after a stroke, but they express that their need for professional empowerment and self-management support are unmet, especially after discharge [16, 17].

Although there is a growing focus on self-management and facilitating an active life after stroke [10], there is still a lack of interventions that would span the whole rehabilitation spectrum – from hospital to municipality to the home of the stroke survivor. Furthermore, the long-term effects of a patient-centered intervention after stroke with a focus on maintaining an active lifestyle, or to build a life has, to our knowledge, only been investigated sparsely.

The objectives of this study are:

- to investigate the effect on self-efficacy, activity, participation, autonomy, and quality of life of a novel self-management intervention supporting elderly people with stroke, from the hospital to the patient’s own home;
- to assess the intervention with regard to informal caregiver burden.

## Methods/Design

### Design

The current study is a randomized controlled trial (RCT), comparing a novel self-management intervention to conventional neurorehabilitation. Baseline assessments will be conducted two weeks before discharge from the hospital, followed by assessments at the three- and nine-month follow-up. Assessors will be blinded to group allocation.

### Concept

The self-management intervention is characterized as a complex intervention – as described by the UK Medical Research Council (MRC) guidelines [18]. Accordingly, the intervention has been developed, feasibility-tested, and evaluated before implementation into the RCT.

### Patient population

We plan to recruit 70 individuals with stroke in the study. All patients with stroke aged > 65 years discharged from the participating specialized neurorehabilitation hospital/center (four different wards from the same hospital and two different wards from an in-care municipality center, who share the same overall philosophy in relation to offer neurorehabilitation) and living in the participating Danish municipality will be considered for inclusion. They will be offered participation in the study if they fulfil the following eligibility criteria:

- Admitted to hospital because of brain infarction or brain hemorrhage;
- No severe cognitive impairment, defined as < 17 on the Montreal Cognitive Assessment (MoCA);
- Able to speak and understand Danish;
- Discharged to own home.

### Randomization

An independent, centralized randomization database will provide allocation concealed to the involved clinicians and assessors. A stratified block randomization of severity of impairment based on Modified Rankin Scale (mRS) (< 3) will be performed within the rehabilitation hospital and the in-care municipality center.

### Intervention

Participants in both groups will receive conventional municipal neurorehabilitation – treatment as usual (TAU). The self-management intervention consists of an add-on intervention to TAU. It has been developed, feasibility-tested, and evaluated using the MRC Framework for Complex Intervention [18]. The first contact will be approximately two weeks before discharge, at the participating specialized neurorehabilitation hospitals/center. This time frame was chosen because most patients are halfway through their course of rehabilitation and municipal rehabilitation is planned. Randomization of patients at this timepoint leaves enough time in the hospital/center to plan and convene in an introduction meeting with patient, caregivers, and the therapist delivering the intervention. After discharge, the intervention will be delivered for nine months, with about eight sessions of a duration of 45–60 min. To allow for flexibility, the number and duration of sessions will be adjusted to individual needs. The self-management intervention will be provided by mentors, a physiotherapist, and an occupational therapist. They have been designated based on their special knowledge about communication and coaching and their experience with the target group.

Patients randomized to the add-on intervention will receive behaviorally focused self-management support designed to increase self-efficacy, quality of life, as well as participation and autonomy. The intervention contains four sub-items: (1) the introduction meeting before discharge: the focus is to establish a good relation by gaining insight into the stroke individuals and their informal caregivers’ lives before the stroke; (2) the municipal rehabilitation with a focus on self-training and self-activation under supervision, plus mapping patients’ social network; (3) supportive meetings/visits or telephone calls, when the municipal rehabilitation has finished: the focus is exclusively on growth and development regarding self-management of activity level, self-efficacy, social network, and quality of life; and (4) pedagogical supporting tools. The patients will have the opportunity to borrow a tablet computer before and after discharge from the hospital. The tablet is meant to support communication and personal relations with the therapist in the municipality and the patients’ network. Furthermore, the tablet can be used for visual goal-setting exercises and reflections after training.

Throughout the entire intervention, the mentors will coach the stroke participants and their informal caregivers and encourage them to be active in decision-making. The intervention has four main purposes: (1) to support the participants in self-management of everyday and leisure activities;

(2) to support them in involving their social networks and interactions in social contexts; (3) to support them in self-assessed activity, self-efficacy, and quality of life; and (4) to support close relatives in maintaining an active life without burden.

Patients in the control group receive individually tailored, standard municipal training, based on the stroke individual’s and their informal caregivers’ preferences, resources, and needs. The municipal neurorehabilitation center receives a rehabilitation plan when the stroke individual is discharged from hospital. This is the starting point for the municipal neurorehabilitation. Goal setting will be based on the Canadian Occupational Performance Measure (COPM). The stroke individuals and their informal caregivers are offered an initial meeting at their own home, plus a meeting in the middle and at the end of the rehabilitation. The duration of the municipality-based neurorehabilitation is individual, but typically lasts 2–3 months after discharge, with 2–3 weekly sessions.

### Outcomes

The primary outcome measure is the Stroke Self-Efficacy Questionnaire (SSEQ), assessing self-rated confidence in performing tasks that may have been affected by stroke [19, 20]. Secondary outcome measures comprise Impact on Participation and Autonomy (IPA) to assess person-perceived restrictions and satisfaction with participation [21–23]. Quality of life is assessed by the short version of Stroke-Specific Quality of Life Scale (SSQoL-12) [24]. The burden on informal caregivers will be assessed by the Caregiver Burden Scale (CBS) [25]. Furthermore, physical activity is assessed using accelerometers.

All assessments, apart from IPA, will be conducted at baseline, three months, and nine months after discharge. IPA will be conducted at three and nine months after discharge. Baseline assessments are performed approximately 14 days before discharge. Assessments at three and nine months are performed in the participants’ homes. Should patients be readmitted to rehabilitation or hospital, follow-up assessments will be conducted at those sites if the patients’ condition allows it. A manual for assessment has been developed and the outcome assessors have been trained to ensure standardized measurements and they are blinded in regards of the randomization.

**Fig. 1:**
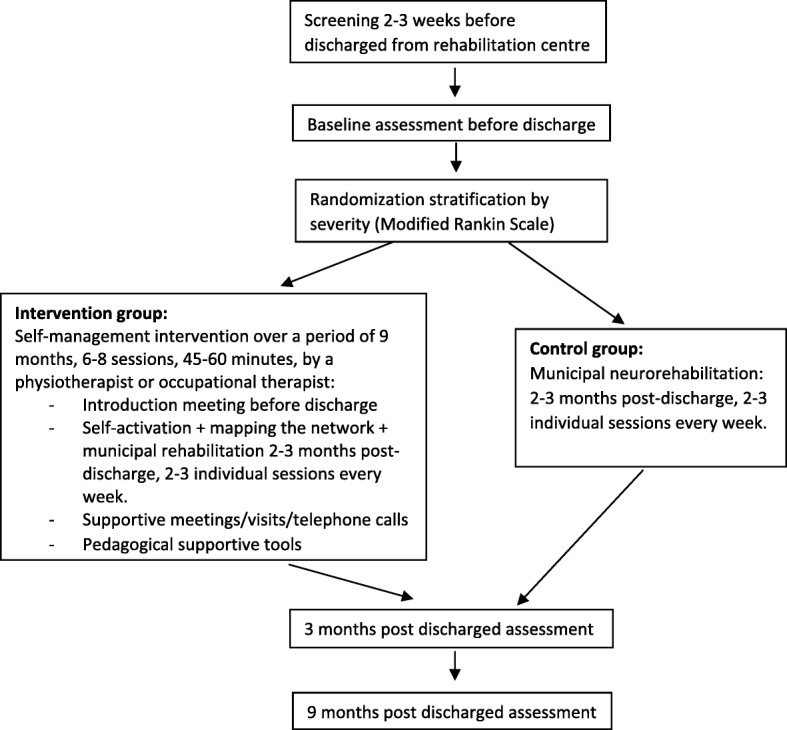
*Flow chart* of patients through the study

Patient, informal caregiver, and therapist satisfaction will be evaluated by way of questionnaires and interviews (Fig. 1).

### Sample size

It is estimated to be realistic to recruit 70 stroke individuals in this study, over a period of 20–30 months, given the number of stroke individuals aged > 65 years discharged to the participating municipality. Earlier research suggests that the SSEQ is appropriate to assess the effect of self-management interventions [26]. The sample size calculation in the present study is based on a feasibility study [26]. The feasibility study revealed a non-significant effect in mean difference between the intervention and control group of 1.91 points on the SSEQ. However, no estimate of the variation in group mean difference from baseline to 12-week follow-up or *p* value was specified. It is therefore not possible to calculate the exact standard deviation (SD) and power for our population. The estimated SD in the present study is therefore a best guess based on the assumption that the SD of the difference will be lower than the SD at baseline and nine-month follow-up, respectively (e.g. 9 points in the study by Jones et al.). Randomization groups are assumed to be equal in size and the significance level is set to 5%. We expect the mean difference from baseline to nine-month follow-up between the intervention and the control group to be higher compared to the study by Jones et al. due to a longer follow-up period and anticipate as many as 14 patients (20%) being lost to follow-up from the originally 70 recruited patients. The estimated final population consist of 56 patients. To elucidate potential implications of the uncertainty of the SD and mean difference after nine months, we have shown iterations of power calculations in Table 1.

**Table 1:** Iterations of mean difference and standard deviations (SD). Estimates in cells are the calculated power given equal sample sizes in control and intervention group and common SD

Power/ %		Mean difference/points			
SD/points	1.5	2	2.5	3	3.5
1.5	96	100	100	100	100
2	79	96	100	100	100
2.5	60	**84**	96	100	100
3	45	69	86	96	100
3.5	35	56	75	88	96

Iterations are based on a sample size of 56 patients allowing for a loss to follow-up of 20%. The power calculation closest to the feasibility study’s estimate of effect is marked in its cell in bold

Note: effect size iterating from 1.5 points to 3.5 points in 4 steps of 0.5 points. The standard deviation is iterated from 1.5 points to 3.5 points in 4 steps of 0.5 points

### Statistical analysis

Group differences using intention-to-treat and per-protocol analyses will be conducted.

Subgroup analyses based on stratification of severity will be performed.

### Study organization

This study is organized and coordinated by the research unit at Hammel Neurorehabilitation Center. The participating stakeholders in this study are the Neuro Center, the Health and Care Center Vikaergaarden in the municipality of Aarhus, and the patient organization Hjernesagen. All parties are participating in the steering committee and are involved in discussing interim results and making the final decisions of the trial.

## Discussion

Self-management interventions are promising strategies to facilitate participation and autonomy. The introduction of a self-management intervention in combination with conventional neurorehabilitation is expected to enhance self-efficacy, activity, participation, autonomy, and quality of life, and to lessen burden on relatives after stroke.

In Denmark, the majority of people aged > 65 years are no longer a part of the labor market [27, 28]. Elderly people with stroke may be an especially vulnerable group with regard to social reintegration because of their reduced personal networks [6, 9]. Self-management is a skill that is likely to differ between elderly and other age groups [29]. Therefore, it seems relevant to investigate what kind of self-management support could be relevant for this group. However, only a few studies have had a specific focus on elderly stroke individuals’ social context in the recovery process [10]. Introducing self-management strategies that are targeted to the elderly and their social context may contribute to a more fulfilling life after stroke.

The self-management intervention in this study intends to facilitate a permanent behavioral change that affects the individual’s ability to cope with the new situation after stroke [12]. This is distinct from other studies which equate self-management with education, skill training, or to increase compliance with recommended treatments [29–31]. The introduction of a self-management approach focusing on behavior change and context-specific strategies within existing stroke rehabilitation is probably an effective way to meet the needs of elderly stroke individuals and help them reintegrate into society.

Cognitive impairments are common after a stroke, either as co-morbidities or as a result of the incident. Measured by MoCA, the prevalence of cognitive impairment has been reported to be about 57% six months after stroke [32]. The relatively low cut-off of 17 points on MoCA allows for a wide range of stroke individuals to be included in this study. The enrolment of participants with mild to moderate cognitive impairment will enhance the external validity of the study.

The cross-sectoral cooperation between the participating neurorehabilitation hospitals/center and the municipal neurorehabilitation center will increase the generalizability of the study results. Since the rehabilitation hospital and the in-care municipality center share the same treatment philosophy along with the fact that the intervention and the control treatment primarily is conducted through the same municipal services, we expect clustering effects within each rehab center/hospital to be small. Furthermore, this trial will provide further evidence of self-management strategies to clinicians, patients, and health economists.

### Trial status

**Fig. 2:**
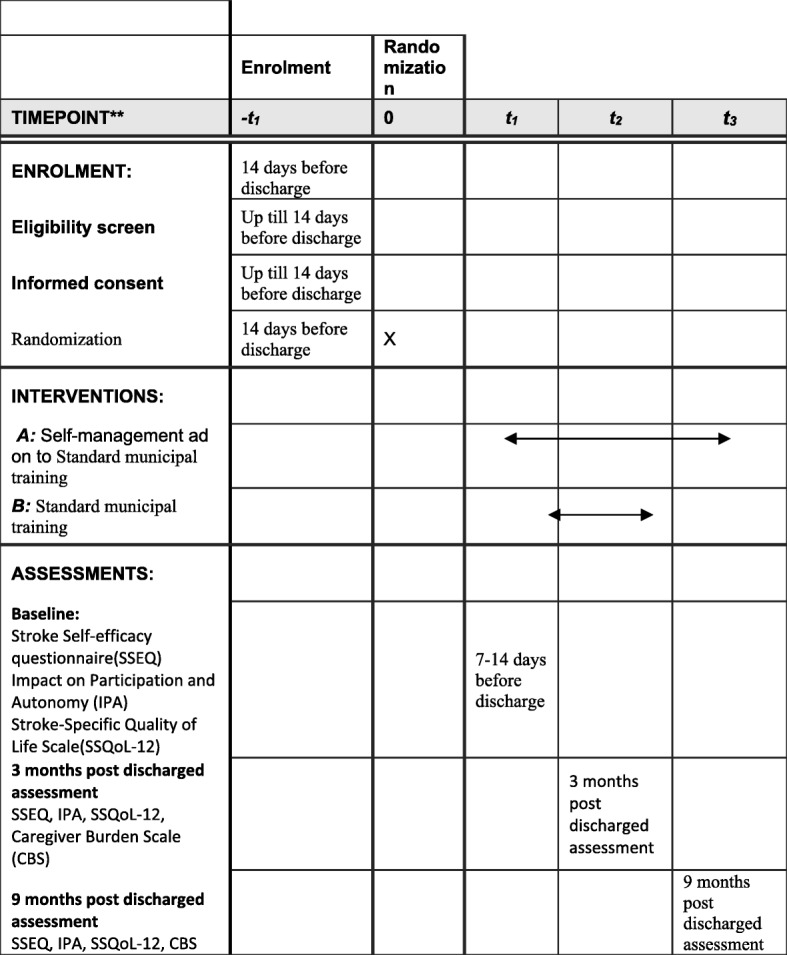
Template of recommended content for the schedule of enrolment, interventions, and assessments

Enrolment of participants started on 15 June 2017. Recruitment, follow-up assessments, and data analyses are expected to be completed by the end of May 2020. See Template Fig. 2 and the Trials populated SPIRIT checklist (Additional file 1).

## Additional file

Additional file 1: SPIRIT 2013 Checklist: Recommended items to address in a clinical trial protocol and related documents*. (DOC 121 kb)

## Abbreviations

CBS: Caregiver Burden Scale
COPM: Canadian Occupational Performance Measure
IPA: Impact on Participation and Autonomy
MoCA: Montreal Cognitive assessment
mRS: Modified Rankin Scale
SSEQ: Stroke Self-Efficacy Questionnaire
SSQoL: Stroke-Specific Quality of Life Scale
UK MRC: United Kingdom Medical Research Council
